# Power sources among district health managers in Ghana: a qualitative study

**DOI:** 10.1186/s12875-022-01678-y

**Published:** 2022-04-04

**Authors:** Vitalis Bawontuo, Augustine Adomah-Afari, Roger A. Atinga, Desmond Kuupiel, Irene Akua Agyepong

**Affiliations:** 1 Department of Health Services Management and Administration, School of Business, SD Dombo University of Business and Integrated Development Studies (SDD-UBIDS), Bamahu- Wa, Ghana; 2 Research for Sustainable Development Consult, Fiapre, Sunyani, Ghana; 3 School of Public Health, University of Ghana, Accra, Legon, Ghana; 4 Department of Public Administration and Health Services Management, Business School, University of Ghana, Accra, Legon, Ghana; 5 Department of Public Health Medicine, School of Nursing and Public Health, University of KwaZulu-Natal, 4001 Durban, South Africa; 6 Ghana Health Service, Accra, Ghana

**Keywords:** Power, District health system, District health managers

## Abstract

**Background:**

In Ghana district directors of health services and district hospital medical superintendents provide leadership and management within district health systems. A healthy relationship among these managers is dependent on the clarity of formal and informal rules governing their routine duties. These rules translate into the power structures within which district health managers operate. However, detailed nuanced studies of power sources among district health managers are scarce. This paper explores how, why and from where district health directors and medical superintendents derive power in their routine functions.

**Methods:**

A multiple case study was conducted in three districts; Bongo, Kintampo North and Juaboso. In each case study site, a cross-sectional design was used to explore the research question. Purposive sampling technique was used to select study sites and 61 participants for interview and focus group discussion. A total of 11 interviews (3 in each district and 2 with deputy regional directors), and 9 focus group discussions (3 in each district) were conducted. Transcriptions of the voice-recordings were done verbatim, cleaned and imported into the Nvivo version 11 software for analysis using the inductive content analysis approach.

**Results:**

The findings revealed that legitimacy provides formal power source for district health managers since they are formally appointed by the Director General of the Ghana Health Service after going through the appointment processes. These appointments serve as the primary power source for district health managers based on the existing legal and policy framework of the Ghana Health Service. Additionally, resource control especially finances and medical dominance are major informal sources of power that district health managers often employ for the management and administration of their functional areas in the health districts.

**Conclusions:**

The study concludes that district health managers derive powers primarily from their positions within the hierarchical structure (legitimacy) of the district health system. Secondary sources of power stems from resource control (medical dominance and financial dominance), and these power sources inform the way district health managers relate to each other. This paper recommends that district health managers are oriented to understand the power dynamics in the district health system.

## Background

Health systems functions with diverse health professionals responding to a range of healthcare needs across populations. Healthcare is routinely delivered by a combined efforts of clinicians and non-clinicians who function with their own set of cultures, norms, educational backgrounds and identities [1]. Everyday interface between and across different health professionals is characterised by power relations that define managerial functions and healthcare delivery. Power play and power sources have thus become fundamental to understanding how health systems function to achieve equity, responsive, effective and efficient care [2]. Power has been variously defined but can simply be described as the ability or capacity to direct or influence a person’s behaviour or act in a way to bring about changes in an event or outcome [2].

Literature has acknowledged that managerial and leadership practices in health systems are characterised by structural, economic and social power dynamics which shapes how people behaviour, act or make choices [3]. But how do individuals gain power over others within the social structures of health systems and organisations? Answers to this question are embedded in how health systems are organised and managed, as well as the divergent sources of power discussed in the literature [3]. This study employed and analysed power from the perspective of French and Raven’s [4] typology of power (cohesion, legitimate, rewards, reference and expert), Finkelstein’s sources of power (structure, ownership, expert and prestige) [5] and Lucio’s theory of resource control [6].

Health systems are generally structured and managed in a top-down approach and this provides incentives for power play. Such power dynamics manifest greatly in decentralised systems where power, authority and resources are often devolved to peripheral units by the central administration [7]. In sub-Saharan Africa, decentralisation is a common reform implemented to enable district health systems to function effectively in improving equity, efficiency and meeting the health needs of peripheral populations [8]. Given the complexity of health systems, decentralisation is widely seen as a vehicle of promoting decisional control of district health managers to navigate routine challenges confronting the provision of health services [9]. With bottom-up decision making approach, decentralisation is argued to lead to better coordination, planning, greater participation in health and increased power for sub-national managers [3]. However, questions abound as to whether decentralisation actually results in achieving intended outcomes such as better population health and managerial efficiency. Too often, the discretionary exercise of power and authority by decentralised health managers tend to stifle quality healthcare delivery or negatively shape the way in which policies and programmes are executed leading to poor outcomes. Yet, an understanding of how and from where decentralised health managers draw on power and exercise same for their own gains is poorly understood empirically. This knowledge gap occasioned this study.

In Ghana, studies have highlighted how micro health managers variously drive power [9, 10]. An important source of such power is linked to the existing leadership hierarchies embedded in decentralised systems. In every district health system, the district director and the medical superintendent play complementary roles in the day-to-day administration of the district health system. The district director who can be a clinician or non-clinician has oversight responsibilities over resource allocation (human, financial, materials), supervision, and project and programme coordination [11]. Generally, the district director presides over all managerial aspects of the district health system and reports to the regional director of health services in the line of duties [12]. The medical superintendent, who is a medical doctor, heads the district hospital which is the referral point for lower level facilities. The medical superintendent coordinates, controls, directs and plans health service delivery in the district hospital. Both the district director and medical superintendent are members of the district health management team (DHMT) – which is the highest decision body on health issues in the district. The complexity of the functional and professional differences between the director and medical superintendent often invoke implicit and explicit power dynamics in determining who gets what power and how [8, 10]. These power dynamics span from routine reporting channels, negotiations, and interaction to decision space on resource allocation and distribution [8].

In principle, for example, the medical superintendent reports to the district director, but in practice, it is not strictly followed especially when the latter is not a doctor [13]. This is partly attributed to the medicalisation of healthcare, giving doctors strong control and dominance over other health professionals [14]. The district director in turn can constrain the functions of the medical superintendent by failing to provide requisite medical and human resources to support care delivery at the district hospital. Given the complex adaptive nature of health systems in general and micro health systems in particular, power play and competition over power can considerably stifle progress in achieving target health incomes. Identifying and dealing with the mechanisms in which district health managers draw on and exercise power is crucial to promote a healthy relation for efficient public health management. In addition, an understanding of how and why social and political factors interact to shape maldistribution of resources in district health systems requires exploring and analysing the sources that give rise to power imbalances and privileges among district health managers. Accordingly, this study sought to explore how and why district directors and medical superintendents derive power in the discharge of their duties.

## Methods

### Study design and participants

The study used multiple case study design involving 3 districts selected from the northern (Bongo district), middle (Kintampo North district) and southern (Juaboso district) belts of Ghana. A ‘case’ is defined as a complex functioning unit, investigated in its natural context [15]. Cross-sectional data collection was implemented in each study site. Within each district, the target participants were health managers (Deputy Regional Health Directors - Clinical Care, district health management team members, hospital management team members and sub-district health leaders) and frontline providers: sectional heads and hospital ward in-charges (nurses, midwives, lab. technicians, health information officers, physician assistants), and midwives, nurses, lab technicians, records officers and dispensing technicians in health centres. All the 3 district health directors sampled were nurses with public health background.

### Sampling

The 3 regions sampled purposively based on the 3 ecological belts of the country and health sector performance indicators. The health sector performance reports described the selected regions in each belt as best performing [16]. We used district health systems that have a district health directorate, a district hospital and a functioning health centre. Bongo district was used as a nucleus district, while Kintampo North and Juaboso districts served as triangulation districts [17]. Bongo-Soe health centre was selected in the Bongo District, New Longoro health centre in the Kintampo North Municipality and Bonsu-Nkwanta health centre in the Juaboso District. The health managers and frontline providers were sampled purposively because of the critical roles that they play in providing health services. A summary of the study sampled participants in the 3 districts is presented in Table 1.

**Table 1:** Summary of study participants showing their categories and districts/regions

Study Site	Health managers		Frontline Healthcare Providers		
	Male	Female	Male	Female	Total
Bongo District/Upper East Region	6	4	10	5	25
Kintampo North / Brong Ahafo Region	7	3	9	5	24
Juaboso district/Western Region	7	2	8	6	23
**Total**	**20**	**9**	**27**	**16**	**72**

### Data collection

Interviews and Focus Group Discussion (FGDs) were used to collect data. Eleven (11) in-depth interviews involving 3 district directors, 3 medical superintendents, 3 sub-district leaders and 2 deputy regional directors - clinical care; were conducted. Also, nine (9) FGDs in each case study site, were conducted. The FGDs were conducted among selected district health managers, frontline providers in the hospitals and health centres. An interview guide with probes and prompts on power sources and power relations was used to collect data across the selected districts. Interview and FGD dates, times and meeting venues were pre-arranged with the participants in order to ensure commitment. All interviews were tape recorded alongside note taking.

### Data analysis

Interviews notes and tape recordings were transcribed verbatim by the authors. The transcripts of the voice-recordings and field notes were cleaned and imported into the Nvivo (version 11) software. The analysis was done using the inductive content analysis approach by coding to identify common patterns [18]. Common patterns in the dataset were categorised into two major themes: formal sources of power (sub-themes were; legitimate source of power for district directors, and legitimate source of power for medical superintendents), and informal sources of power (sub-themes were; financial dominance and medical dominance).

## Results

The results are structured around the themes that emerged from the data analysis. These themes are related to the formal (legitimate) as well as informal power sources that define how district directors and medical superintendents exercise power, with whom and over who, and why.

### Legitimate source of power - district directors

Even though there are relational gaps in the district health system, the findings revealed that district directors are still recognized as formal heads of the district health systems and derive powers from their legitimate positions. Legitimate power is derived from the position a person holds in an organization’s hierarchy. Thus, the study found that district directors gain power because district health directorates are the apex of all health institutions at the district health system. As to why district directors have legitimate power, the findings revealed that they serve as figureheads and represent the district in various meetings. For instance, they represent the health sector during district assembly meetings – a meeting of assembly members and heads of various institutions in the district - to discuss developmental issues of concern. However, the medical superintendent or hospital administrator represents the health sector in such assembly meetings in the absence of the district director:

[…] If I (district director) am not able to attend any district assembly meeting, the medical superintendent or the hospital administrator attends on my behalf and brief me afterwards. […] **(Interview, District Director).**

In such meetings, the district directors have power to negotiate and dialogue with the assembly, thus pushing the entire district health agenda for consideration by the assembly.

The findings also revealed that district directors represent the district during regional health management team meetings. Regional health directorates provide managerial support to the district health services, and periodically hold management meetings involving all districts in the region to discuss healthcare delivery issues. Additionally, regional health directorates organise periodic performance review meetings to monitor the performance of the districts. In such meetings, district directors present a composite report of all health activities carried out at the district hospital, health centres and the CHPS zones/compounds during these regional performance review meetings:

[…] The district is preparing for the annual regional performance review meeting, and the district director will lead the team to present the district annual activities in one composite report […] **(Interview, Medical Superintendent).**

This clearly shows that the district director derive power as a legitimate head of the district; present and defend healthcare activities for the year under review and set targets for the next year.

### Legitimate source of power - medical superintendent

It was found that medical superintendents derive power from their positions as heads of the district hospitals. A review of the Ghana Health Service and Teaching Hospitals Board Act 525 (1996) showed that district hospitals have financial and administrative authority to operate as independent budget management centres (BMCs) within the district health systems. With this policy, district hospitals are devolved from the district health directorates and have the power to make decisions for the smooth functioning of the hospitals. This demonstrates how medical superintendents gain legitimate power to oversee and supervise district hospital activities. Consequently, even though the organisational structure of district health systems shows that district directors head all health institutions, participants reported that district hospitals and district health directorates have separate heads:

[…] The district director is the head of the district health directorate, and the medical superintendent is the head of the district hospital, which is part of the district health directorate […] **(Interview, Deputy Regional Director, Clinical Care).**

Another reason for which medical superintendents acquire legitimate power was that district hospitals are autonomous and deal directly with the Regional Directors of Health Services in spite of the fact that district hospitals are integral part of the district health systems. This was confirmed in this statement:

[…] Theoretically, the district hospital is part of the district health directorate, but practically or in reality it is not […] In terms of management, the hospital does not report to the district health directorate, but directly to the regional director […] **(FGD, Nurse).**

Consequently, the annual performance appraisals of medical superintendents are not done by the district directors, but by their respective regional directors of health services. As pointed out by this participant:

[…] Though the hospital is an integral part of the district, I (district director) do not appraise him (medical superintendent); we are both appraised by the regional director every year […] **(Interview, District Director).**

While the established policies prescribed the formal power relationships between the district director and the medical superintendent, there were perceived conflicts due to the kind of informal sources of power that exist in practice. Such informal power sources also inform how these district health managers relate to each other within the district health system.

### Informal power sources of district health managers

The findings further revealed that the availability and control of resources (finances) as well as knowledge and expertise were additional layers of legitimate power of the district health managers. These are further explored below.

#### Financial dominance as an informal power source

The study participants attributed this form of power to government’s inability to directly finance district health activities through subventions. Instead, government is funding district health activities through funds generated internally at the point of care. This means that district health institutions must maximise the use of their resources in order to generate enough funds to ensure sustainability. For this reason, district health institutions that generate funds, and are able to generate more, have access to finances to support and sustain their healthcare activities:

[…] Budgetary allocations to the BMCs have dwindled or stopped completely […] Government is now funding our budgets through the services we provide […] So we just have to generate more money to survive […] **(FGD, Nurse).**

Medical superintendents derive financial power by virtue of their internally generated funds (IGF) and make independent decisions on the use of financial resources without consulting the district directors. The study participants disclosed that district hospitals provide services that are paid for either at the point-of-care (cash and carry) by the service recipients, or retrospectively by the National Health Insurance Scheme (NHIS). In this instance, medical superintendents have strong control over financial resources generated through the services delivered at the hospital:

[…] District hospitals generate funds from their services, and as medical superintendents, we have authority to make independent decisions on the funds we generate […] Yes, we have that power […] **(Interview, Medical Superintendent).**

On the contrary, the district health directorates do not generate any revenue and solely depend on government subvention. This is because district health directorates, per the policy guidelines of the Ghana Health Service provide preventive health services which are not income generating. This poses financial challenge in the day-to-day administration of the district health directorates. At the same time, financial support from government has dwindled leaving directorates with budgetary deficits:

[…] We do not generate any income from the directorate here, we normally depend on central government subventions to carry out our planned activities, but these days we hardly get money from the central government […] **(Interview, District Director).**

Furthermore, the district directors rely on the hospitals for financial support to implement health and administrative activities at the district health directorate levels. Study participants confirmed that district directors receive various forms of support (including traveling expenses, financing health programmes and administrative support) from the medical superintendents. This financial support is mandatory, and can be accessed in different forms contingent on the needs of the directorates:

[…] There was a letter asking hospitals to be supporting the district health directorate […] What we normally do here is that, anytime we are in need of something and we are tight; we call on them (hospital management) for support […] **(Interview, District Director).**

Even though the support system is mandatory, study participants reported that some medical superintendents are reluctant to comply with directive. This implies that implementation of the directive depends on the relationship between medical superintendents and district directors, and how much district directors can negotiate for their packages. This confirms why medical superintendents have so much resource power over the district directors.

#### Medical dominance as an informal power source

The findings revealed how and why medical dominance serves as another source of power for district health managers. For instance, it was reported that medical doctors use their knowledge and expertise to gain power over other health professionals in the health sector. This suggests that medical doctors derive power from their training and profession. Consequently, it was suggested that medical doctors should be positioned higher in the hierarchical ladder of any health system. This suggests that no other health professional can exercise power over the medical doctor:

[…] The districts should be manned by medically trained persons, meaning persons who have gone through medicine and passed out […] The post/position should be a preserve for doctors, so that all the other categories of health professionals can fall in place without any qualms […] **(Interview, Deputy Regional Director, Clinical Care).**

A review of the GHS job description and specification for medical superintendents and district directors confirmed that medical superintendents are medical doctors (preferably specialists), and district directors are either medical doctors (with public health background) or any other health professionals who have public health background. One of two scenarios, therefore, exist in a district. Firstly, a district director who is not a medical doctor works with a medical doctor as the medical superintendent. Secondly, a district director who is a medical doctor works with a colleague medical doctor as the medical superintendent in the district hospital.

The findings revealed that in the first scenario where the medical superintendent is a medical doctor and the district director is not, the former derives power and exercise same over the district director. Participants reported that in districts where a district director (who is not a medical doctor) attempts to show superiority over the medical superintendent, he/she often faces challenges:

[…] If the district director thinks that he/she is a boss to somebody like the doctor or the medical superintendent, definitely he/she will run into problems because when you compare a lot of things, it cannot be so […] **(Interview, Medical Superintendent).**

Another participant indicated:

[…] Some doctors do not think that a public health nurse or a disease control officer or any other health professional should be a district director and ask them to do things; they will think the person is lording over them […] **(Interview, Deputy Regional Director, Clinical Care).**

In the second scenario where both actors (district director and medical superintendent) are medical doctors, it was reported that medical dominance was non-existent to some extent. The district director relates to the medical superintendent as a colleague, supporting and coordinating each other’s efforts to ensure quality healthcare delivery:

[…] If the district director is a medical doctor, you see each other as colleagues […] When you are hot he comes in to help you, if you are travelling, he comes and takes over from you; the cooperation is better with a colleague medical doctor as a district director […] **(Interview, Medical Superintendent).**

Nonetheless, in the absence of medical dominance, as in the case of colleague medical doctors working together as district director and medical superintendent in one district, the issue of who has power, and who has control over who still exist:

[…] Even the doctor-doctor scenario, they quarrel, they do not accept instructions from each other […] **(Interview, Deputy Regional Director, Clinical Care).**

## Discussion

This study explored how and why district health managers, such as district directors of health services and district hospital medical superintendents, gain what power, over whom, and to what extent, in the discharge of their duties. The study employed cross-sectional study design in a multiple case study involving 3 districts in Ghana. The findings revealed that district directors and medical superintendents have diverse sources of power including legitimacy, medical dominance and financial dominance, and these power sources inform how district directors and medical superintendents relate to each other in the discharge of their duties.

The findings revealed how legitimacy serves as power source for both district directors and medical superintendents since they are formally appointed by the Director General of the GHS after going through the appointment processes. These appointments serves as the primary power source for district health managers based on the existing legal and policy framework of the GHS. Legitimacy is identified as power source which gives appointed officials the right and authority to perform their job roles within the formal organisational arrangements [4]. Thus, as some authors acknowledged, this source of power is a formal and positional power, which is vested in the office rather than the person [19, 20]. This power source presupposes that the legitimate power of medical superintendents is at par with that of district directors, introducing a horizontal power structure which potentially distorts the hierarchical power structure of GHS at the district level.

By legitimate power, it was evident that both district directors and medical superintendents have additional powers such as coercive and rewards. For instance, district directors and medical superintendents appraised and recommend their staff for promotion to higher positions, implying they have rewards power. However, from the findings, district directors exercise coercive, rewards and referent power at the district health directorate level, which includes the sub-districts and CHPS zones, but not at the district hospital level. Thus, staff of the district hospital are not likely to recognise and accept the district director as legitimate head, a situation district directors are worried about. This is in spite of the fact that district hospitals are integral parts of district health systems where district directors are expected to be the overall heads of the district health services. These findings conform to the literature that the management and services of district hospitals are separated from the management and services of Primary Health Care (PHC) institutions [21, 22].

Medical superintendents on the other hand exert their powers over only staff of district hospitals and may not be able to extend these powers to the staff of health centres and CHPS zones. Yet, an interesting finding of this study relates to the fact that health centre staff recognise district hospitals as superiors and constantly discuss medical issues with medical superintendents and their staff. This implies that medical superintendents have expertise and potentially draws some referent powers from the health centre staff. Thus, as compared to district directors, medical superintendents potentially have enough powers from the perspective of the health centre staff. This poses as a threat to district directors as persons who directly manage the sub-districts. This is likely to introduce power struggle between district directors and medical superintendents and has the potential to affect the organisation and delivery of clinical services at the health centre level.

Adding to the layer of complex horizontal power relations between district directors and medical superintendents are various informal power sources such as medical dominance and financial dominance. It was clear that knowledge and expertise serve as power source leading to the concept of expert power [4]. This was evident in the findings since the medical profession is often perceived as the giant of the health sector workforce. medical dominance was thus, a prominent informal source of power, and contributed significantly to the distraction of formal power structure in the district. It is in this light that this study revealed that medical superintendents exert expert power over district directors who are not medical doctors. This is consistent with the position of a study which argued that doctors use their powers as experts to influence other health professions [23].

In Ghana, medical doctors are very prominent in the health sector, and occupy most key positions in the health sector. For instance, in typical healthcare settings like the hospital, the medical doctor, irrespective of his/her experience, assumes the most senior position as the medical superintendent. The findings are also consistent with the findings of another study which indicated that medical doctors are dominant and influential in healthcare organisational management [24]. This probably explains why many managerial positions in the health sector are filled with medical doctors. This also suggests that medical doctors are the ultimate decision-makers, both at the service provision and management levels.

It was widely reported that medical doctors are unable to work under, and/or accept instructions from other health professionals, and therefore, have dominion over all other health professionals. This finding corroborates the literature that medical dominance was considered a structural barrier to health services delivery, posing workplace dissatisfaction among nurses [25]. Similarly, the findings agree with another study about mobile health teams in the Brazilian health system, where there was dominance of the medical profession over the other health professions leading to constrained functions [26]. The authors concluded that the medical dominance disrupted service provision as some team members felt disregarded by the medical professionals on some occasions.

The literature argues that the supremacy of medicine as the scientific framework gives doctors significant influence over other health professions [6]. In the same way, this study revealed that medical superintendents (who are usually medical doctors) do not accept nurses or other health professionals when appointed as district directors, since they are not colleague medical doctors. This implies that medical dominance puts constraints on effective management of the district health systems, as district directors who are not medical doctors are not able to exercise control over medical superintendents.

However, this trend of having medical doctors in managerial positions is progressively changing in Ghana’s health system. Currently, the position of district directors, which used to be the preserve of medical doctors with public health background [27], has been opened to all health professionals who have public health background. The outcome of this policy implementation contributes to strained power relations between district directors and medical superintendents.

Conversely, the findings also revealed that medical dominance was absent in situations where both district directors and medical superintendents were medical doctors. It was indicated that they regard each other as colleagues having the same expertise, and therefore, unable to exercise superiority based on that. As a result, such circumstance requires both parties to rely on each other’s power source, which could be legitimacy or access to financial resources. The bottom line is that, in most cases where district directors and medical superintendents are both medical doctors, their relationships are more cordial than when district directors have a different background such as nursing or disease control as revealed by the findings of this study.

This study findings revealed that central government subvention to district health institutions has dwindled leaving district directors and medical superintendents with limited control over financial resources. Thus, access to cash and control of same, has significant influence on the power base of the district health managers. District hospital derive financial resources from the NHIS claims repayments. This is consistent with the findings of a study which indicated that NHIS contributes significantly to healthcare financing in Ghana [28]. Financing healthcare through NHIS claims repayments implies that district health institutions that provide services are likely to have and control funds.

Consequently, the study findings revealed that the effects of district health institutions financing their own activity budgets through their Internally Generated Funds (IGF) have left district directors in a vulnerable situation. That is, they have no funds to support their budgets. This implies that district directors have no option, but to depend on the district hospital’s IGF for survival. This finding is consistent with the literature that the district health directorates rely on district hospital’s for financial support [29]. The authors concluded that this dependence makes the district health directorates have less power over district hospitals.

## Conclusions

 The study concludes that both district directors and medical superintendents have legitimate power which reflects a horizontal power structure rather than a hierarchical power structure as depicted by the policies and guidelines of the GHS. Medical dominance and financial dominance are additional sources of power for these district health managers that shape the performance of their duties. The study recommends that district health managers are oriented to understand the power dynamics in the district health system. District directors and medical superintendents need to work together in a horizontal power structure in their routine functions. This effort will facilitate effective support systems for district health development.

## Data Availability

All essential data are within the manuscript. The transcripts are also available upon a request to Dr. Vitalis Bawontuo via bawontuovitalis@yahoo.com.
